# Risikokommunikation zum Schutz vor Gefahrstoffen am Arbeitsplatz

**DOI:** 10.1007/s00103-022-03530-1

**Published:** 2022-04-11

**Authors:** Rüdiger Pipke, Annette Wilmes

**Affiliations:** Fachbereich Gefahrstoffe und Biologische Arbeitsstoffe, Bundesanstalt für Arbeitsschutz und Arbeitsmedizin, Friedrich-Henkel-Weg 1–25, 44149 Dortmund, Deutschland

**Keywords:** Arbeitsschutz, Risikobewertung, Sicherheitsdatenblatt (SDB), Technische Regeln für Gefahrstoffe (TRGS), Einfaches Maßnahmenkonzept Gefahrstoffe (EMKG), Occupational safety, Risk assessment, Safety data sheet (SDS), Technical rules for hazardous substances (TRGS), Easy-to-use Workplace Control Scheme for Hazardous Substances (EMKG)

## Abstract

Gefahrstoffe sind in der Arbeitswelt allgegenwärtig. Beschäftigte aus allen Branchen sind bei ihrer Arbeit mit Gefahrstoffen unterschiedlichen Risiken ausgesetzt. Dies betrifft auch Personen, die nicht unmittelbar mit Gefahrstoffen umgehen (Bystander). Risikokommunikation im Arbeitsschutz spricht verschiedene Zielgruppen an. Das Spektrum reicht vom Groß- zum Kleinunternehmer über Sicherheitsfachkräfte, Betriebsärzte, Betriebs- und Personalräte bis hin zu den Beschäftigten und der breiten Öffentlichkeit. Risikokommunikation im Arbeitsschutz hat das Ziel, entlang von Lieferketten Risikobewusstsein bei Herstellern, Arbeitgebern und den Beschäftigten zu schaffen, risikohaftes Verhalten zu vermindern und risikominderndes Verhalten zu bestärken.

In diesem Beitrag werden Instrumente der Risikokommunikation erläutert und bezüglich ihrer Wirksamkeit betrachtet. Das betrifft verbindliche Instrumente im europäischen Binnenmarkt wie das Gefahrenetikett oder das Sicherheitsdatenblatt (SDB). Auf Ebene der Gefahrstoffverordnung sind es die Technischen Regeln für Gefahrstoffe (TRGS), Grenzwerte, das Einfache Maßnahmenkonzept Gefahrstoffe (EMKG) und als kreatives Instrument die Objekte der „DASA Arbeitswelt Ausstellung“ in Dortmund. Der Beitrag zeigt, dass eine Anpassung der Instrumente besonders für kleine und kleinste Unternehmen notwendig ist, damit diese einen passenden Einstieg in die Risikobewertung finden. Oft sind diesen die vorhandenen Instrumente nicht bekannt, zu umfangreich oder schwer verständlich. Handlungsempfehlungen wären hier hilfreich. Vorliegende wissenschaftliche Studien konzentrieren sich eher auf die Defizite in der Risikobewertung als auf die Entwicklung von effizienten Wegen der Risikokommunikation. Hier sind weitere Analysen der Bedürfnisse unterschiedlicher Zielgruppen für eine adressatengerechte Risikokommunikation erforderlich.

## Abstract (en)

Hazardous substances are ubiquitous in the world of work. Employees from all sectors are exposed to different risks when working with hazardous substances. This also applies to people who do not directly handle hazardous substances (bystanders). Risk communication in occupational safety and health takes place at different levels. The spectrum ranges from large to small companies, safety experts, company physicians, works and staff council representatives to employees and the general public. Risk communication in occupational safety and health along the supply chain aims to create risk awareness among manufacturers, employers and employees to reduce risky behaviour and to encourage risk-reducing behaviour.

Instruments of risk communication are explained in this article and considered with regard to their effectiveness. This affects binding instruments in the European internal market such as the hazard label or the safety data sheet (SDS). At the level of the Hazardous Substances Ordinance, they are the Technical Rules for Hazardous Substances (TRGS), limit values, the Easy-to-use Workplace Control Scheme for Hazardous Substances (EMKG) and, as a creative instrument, objects of the “DASA Working World Exhibition” in Dortmund, Germany. The results of the paper show that an adaptation of the instruments is necessary, especially for small and micro-enterprises, in order to find the appropriate entry point into risk assessment. Often, the existing instruments are not known, too extensive or difficult to understand. Recommendations for action are necessary that make it easy to get started with risk assessment. Existing scientific studies tend to focus on the deficits in risk assessment rather than on the development of efficient ways to communicate risks. Further analyses of the needs of the different target groups are necessary for appropriate risk communication.

## Einleitung

Die Bundesanstalt für Arbeitsschutz und Arbeitsmedizin (BAuA) setzt sich für die Verbesserung von Sicherheit und Gesundheit des Menschen in seiner Arbeits- und Lebenswelt ein. Auf Grundlage der Ergebnisse zahlreicher Studien berät sie die Politik, nimmt gesetzliche Aufgaben wahr und transportiert aufgearbeitetes Wissen in die betriebliche Praxis und die breite Öffentlichkeit. Sicherheit und Gesundheit des Menschen am Arbeitsplatz sind nur möglich, wenn im Betrieb Risiken erkannt und bewältigt werden können. Dazu führen Arbeitgeber Beurteilungen der Arbeitsbedingungen durch und legen zielgerichtete Präventionsmaßnahmen fest. Instrumente der Risikokommunikation helfen Arbeitgebern, ihre Beschäftigten in den unterschiedlichen Arbeitssituationen zu erreichen.

**Figure 1:**
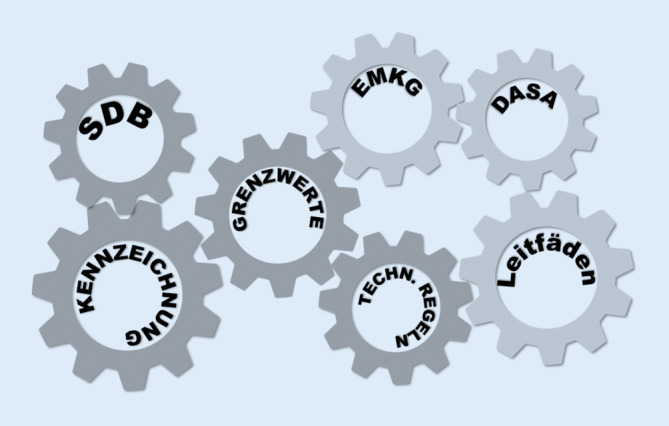

Für die Kommunikation von Risiken durch Gefahrstoffe gibt es zahlreiche Instrumente. Die Autoren beschreiben in diesem Beitrag zum einen Instrumente (wie z. B. Piktogramme), die Hersteller und Importeure den Unternehmen aufgrund ihrer gesetzlichen Verpflichtung zur Verfügung stellen müssen; zum anderen Instrumente, die die Gefahrstoffverordnung [1] konkretisieren und Arbeitgeber bei der Beurteilung der Risiken unterstützen (Abb. 1). Dabei beantworten sie die Frage, wo auf gesetzlicher und betrieblicher Ebene weiterer Handlungsbedarf besteht.

## Bedeutung von Gefahrstoffen für Beschäftigte

Für Beschäftigte hat das Thema Chemikaliensicherheit eine hohe Bedeutung. In Deutschland stehen etwa die Hälfte aller Verdachtsanzeigen auf eine Berufskrankheit und 85 % aller beruflich bedingten Todesfälle im direkten Zusammenhang mit einer Exposition gegenüber Gefahrstoffen [2]. Auffällig sind die hohen Anteile von obstruktiven Atemwegserkrankungen an Staubarbeitsplätzen (18 %), die durch chemisch-irritativ oder toxisch wirkende Stoffe verursacht werden, und Hauterkrankungen (24 %), bei denen es sich überwiegend um irritative Kontaktekzeme handelt [2]. Beide Erkrankungen lassen sich häufig auf nicht gekennzeichnete, entstehende oder freigesetzte Gefahrstoffe zurückführen. Für rund 2600 jährliche Todesfälle sind gefahrstoffbedingte Krebserkrankungen die Ursache. Hierbei spielt immer noch die Exposition gegenüber Asbest eine herausragende Rolle. Relevant sind hier auch die langen Latenzzeiten der Krebsentstehung [2]. Betrachtet man die Ebene der Unternehmen, so geben 11,5 % aller Betriebe in Deutschland an, dass „fast alle“ oder „eher viele“ Beschäftigte von Gefährdungen durch den Umgang mit Gefahr- oder Biostoffen betroffen sind [3]. In der Datenbank zur Einstufung und Kennzeichnung der Europäischen Chemikalienagentur (ECHA) sind zurzeit etwa 200.000 Stoffe gelistet [4]. Während nur ein kleiner Teil der Gefahrstoffe in Verbraucherhände oder die Umwelt gelangt, können Beschäftigte grundsätzlich allen hergestellten, vermarkteten und am Arbeitsplatz entstehenden Gefahrstoffen ausgesetzt sein.

## Risikobewertung im Arbeitsschutz

**Figure 2:**
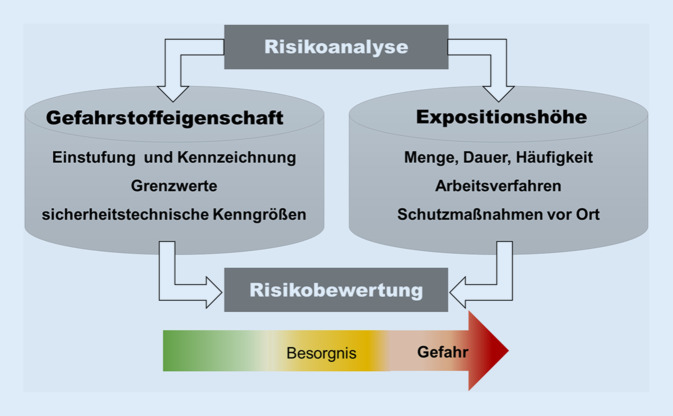

Bei einer Risikobewertung für Gefahrstoffe werden zur Einschätzung eines Gesundheitsrisikos die intrinsische Gefahrstoffeigenschaft und die Expositionshöhe während einer Tätigkeit betrachtet (Abb. 2). Bei Brand- und Explosionsrisiken wird das Risiko eingeschätzt über die Gefahrstoffeigenschaften und die Wahrscheinlichkeit, dass ein Brand oder eine Explosion entstehen kann. Die Risikobewertung weist Handlungsbedarf für technische, organisatorische und persönliche Schutzmaßnahmen aus.

Durch gezielte, oft aufwendige Maßnahmen lässt sich die Exposition gegenüber Gefahrstoffen senken, aber nie ganz ausschließen, da viele natürliche Gefahrstoffe (z. B. Metalle) und synthetische Gefahrstoffe (z. B. Kunststoffe) nicht verzichtbar sind. Einige Metalle und ihre Verbindungen sind in der Europäischen Union (EU) als krebserzeugend eingestuft und gehören zu den Stoffen mit besonderer Besorgnis für Mensch und Umwelt. Dazu gehören krebserzeugende, mutagene und reproduktionstoxische Stoffe. Diese Metalle sind aber aufgrund ihrer Eigenschaften ein wesentlicher Baustein vieler Alltagsgegenstände (z. B. in elektronischen Bauteilen).

Ein anderes Beispiel sind Gefahrstoffe, die während einer Tätigkeit entstehen, z. B. Abgase, Gefahrstoffe aus chemischen Reaktionen oder unbekannte Gefahrstoffe bei Recyclingtätigkeiten. Jüngste Ereignisse, wie die Explosion in einem Tanklager einer Sondermüllverbrennungsanlage im Chempark Leverkusen [5], belegen das Unfallrisiko in der Entsorgungs- und Recyclingbranche.

Weil Gefahrstoffe am Arbeitsplatz nicht wegzudenken sind, ist es notwendig, stets die Expositionshöhe und die Wahrscheinlichkeit eines Unfallereignisses in die Risikobewertung mit einzubeziehen. Denn nur in wenigen Fällen kann der Gesetzgeber diese Gefahrstoffe vom Markt nehmen oder die Industrie solche Gefahrstoffe im Rahmen der Substitutionsprüfung durch weniger gefährliche Alternativen ersetzen. Der Schlüssel hier ist die emissionsarme Gestaltung von Stoffen, Produkten und Arbeitsverfahren, bei denen Beschäftigte z. B. beim Umgang mit wirkstarken, krebserzeugenden Gefahrstoffen nur einem akzeptierten, geringen Gesundheitsrisiko ausgesetzt sind.

## Instrumente der Risikokommunikation

### Rechtlicher Hintergrund

Die Risikokommunikation beginnt beim Import und Inverkehrbringen von Chemikalien und Produkten auf den europäischen Binnenmarkt. Der Hersteller oder Importeur ist verpflichtet, Informationen und Empfehlungen zur sicheren Handhabung innerhalb der Lieferkette weiterzugeben, um somit auch den Arbeitgeber bei seinen Pflichten zu unterstützen.

Chemische Stoffe sind nach der REACH-Verordnung (Registration, Evaluation, Authorisation of Chemicals; [6]) registrierungspflichtig. Hierzu muss der Hersteller verlässliche Daten zu physikalischen und chemischen Eigenschaften sowie zur Human- und Umwelttoxizität generieren. Diese Daten fließen in die Einstufung von Stoffen und Gemischen, in die Ableitung von Grenzwerten und in die Empfehlungen von Schutzmaßnahmen ein. Die wichtigsten Instrumente der Risikokommunikation sind die Kennzeichnung nach der CLP-Verordnung (Classification, Labelling, Packaging; [7]) und das Sicherheitsdatenblatt (SDB). Besonders besorgniserregende Stoffe können einer Zulassungspflicht oder einer allgemeinen Beschränkung unterliegen. Die Risikokommunikation erfolgt dann über das SDB nach Anhang II und den Anhängen XIV und XVII der REACH-Verordnung. Hersteller und Importeure können die sehr unterschiedlichen örtlichen Gegebenheiten in den Betrieben nicht vollständig mit ihren Möglichkeiten abbilden. Daher sind weitere Instrumente der Risikokommunikation notwendig. Dazu gehören z. B. die Technischen Regeln für Gefahrstoffe (TRGS; [8]). Sie werden vom Ausschuss für Gefahrstoffe (AGS), einem mit Sozialpartnern, Aufsichtsdiensten und Forschenden besetzten Beratungsgremium des Bundesministeriums für Arbeit und Soziales (BMAS), entwickelt und vom BMAS bekannt gemacht. Technische Regeln konkretisieren die Gefahrstoffverordnung. Darüber hinaus gibt es zahlreiche Handlungsempfehlungen und Hilfestellungen der Unfallversicherungsträger, der Bundesländer, der BAuA und anderer Institutionen, die Arbeitgeber bei der Risikobewertung unterstützen.

### Risikokommunikation anhand von Piktogrammen

Startpunkt für die Risikokommunikation ist die Kennzeichnung von gefährlichen Stoffen und Gemischen. Jeder kennt die auf der Spitze stehenden weißen Quadrate mit rotem Rand, die in der Mitte ein schwarzes Symbol für eine oder mehrere gefährliche Eigenschaften tragen. Ein Gefahrenpiktogramm (Tab. 1) hat eine direkte Warnwirkung und ist sowohl für Arbeitgeber und Beschäftigte wie auch für Verbraucher unverzichtbar [9]. Es gibt weltweit kein anderes Instrument, das bei allen Betroffenen so bekannt ist und auf Gefahreneigenschaften aufmerksam macht [10].

**Table 1:** 

*Gefahrenpiktogramm*	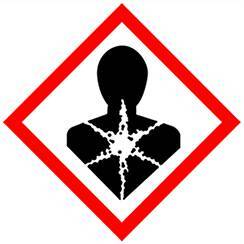	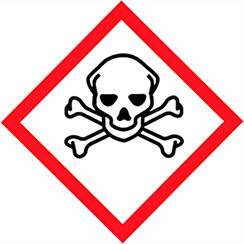
*Symbol*	Gesundheitsgefahr „Torso“	Totenkopf mit gekreuzten Knochen
*Gefahrenklassen*	Sensibilisierung der Atemwege	Akute Toxizität (oral, dermal, inhalativ)
	Keimzellmutagenität	
	Karzogenität	
	Reproduktionstoxizität	
	Spezifische Zielorgantoxizität	
	Aspirationsgefahr	

Neben den Piktogrammen vermitteln weitere standardisierte Elemente auf dem Kennzeichnungsetikett der Verpackung das Ergebnis der Einstufung. Erst in Kombination mit den Gefahrenkategorien, Signalwörtern „Gefahr“ und „Achtung“ sowie den standardisierten Gefahrenhinweisen wird die Eigenschaft der Chemikalie beschrieben.

Die Einstufung, Kennzeichnung und Verpackung von Gefahrstoffen sind in der CLP-Verordnung [7] verbindlich seit 2008 für die EU festgelegt. Diese beruht auf Empfehlungen der Vereinten Nationen (UN), die 2002 als UN GHS (Globally Harmonized System) verabschiedet und 2003 erstmals als sogenanntes „Purple Book“ veröffentlicht wurden [11].

Die Umsetzung der CLP-Verordnung bedeutet bis heute für Hersteller, Importeure, Arbeitgeber, Beschäftigte und Aufsichtsdienste eine große Herausforderung in der Risikokommunikation. Dementsprechend hoch war seitens der BAuA der Aufwand für die Vermittlung der neuen Einstufungs- und Kennzeichnungsvorschriften. Die üblichen Wege der Kommunikation über Veröffentlichungen in Fachzeitschriften, Vorträge, Fortbildungen und Flyer wurden durch weitere Maßnahmen ergänzt. Zum einen wurde eine Serie von 4 Postern aufgelegt; zum anderen wurde mit einer robusten Memocard ein neues Format für die Unterweisung der Beschäftigten entwickelt [12–16].

**Figure 3:**
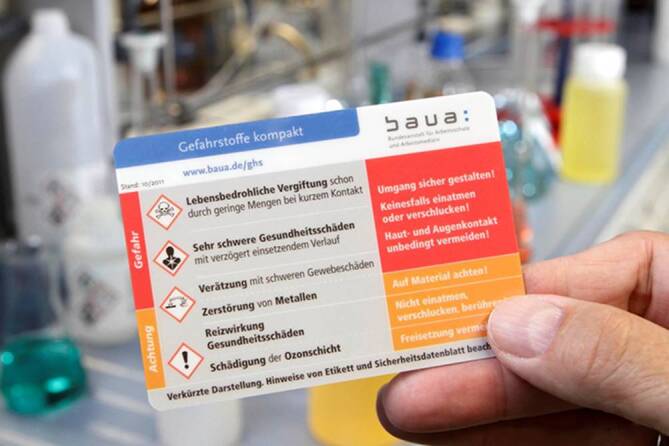

Zielgruppe der Poster sind Arbeitsschutzakteure mit einem Grundwissen über Einstufung und Kennzeichnung. Wie die internen BAuA-Statistiken zeigen, sind die 4 Poster als Kommunikationsmittel insgesamt ein Erfolg. In den letzten 6 Jahren gab es mehr als 400.000 Downloads und 54.500 Printfassungen, die verteilt wurden und immer noch verteilt werden. Leicht verständlich, bis auf die Ebene der Beschäftigten orientiert, ist die Memocard (Abb. 3). Auf Scheckkartengröße werden die wichtigsten Änderungen des neuen Einstufungs- und Kennzeichnungssystems transportiert.

### Das Sicherheitsdatenblatt als wichtige Informationsquelle

Der Hersteller oder Lieferant eines chemischen Produktes ist gemäß REACH-Verordnung verpflichtet, seine Informationen zur sicheren Handhabung über das Sicherheitsdatenblatt (SDB) „frei Haus“ an industrielle und gewerbliche Abnehmer zu kommunizieren [6]. Für den Arbeitgeber und die fachkundigen Personen, die im Betrieb Risiken analysieren und bewerten, ist das SDB die wichtigste Informationsquelle [17].

Relevant sind neben der Einstufung und Kennzeichnung Angaben zu Grenzwerten, zu physikalischen und chemischen Eigenschaften sowie zur Stabilität und Reaktivität. Darüber hinaus werden Empfehlungen zu adäquater Schutzausrüstung, z. B. geeignetem Handschuhmaterial oder Atemschutz gegeben. Für Stoffe, die in Mengen über 10 t pro Jahr in der EU produziert oder importiert werden, müssen Hersteller oder Importeure einen Stoffsicherheitsbericht erstellen. Handelt es sich um einen gefährlichen Stoff (eingestuft nach der CLP-Verordnung) oder um einen Stoff mit einer umweltgefährlichen Eigenschaft, dann müssen Expositionsszenarien mit sicheren Verwendungsbedingungen abgeleitet werden. Sie haben das Ziel, die Exposition der Beschäftigten unter gesundheits- oder risikobezogene Grenzen zu minimieren. Expositionsszenarien werden dann in einem erweiterten Sicherheitsdatenblatt kommuniziert.

Das SDB und die Expositionsszenarien beschreiben allgemein und abstrakt Bedingungen, unter welchen eine sichere Verwendung des Gefahrstoffes möglich ist. Für die Risikobewertung im Betrieb kann dies ein guter Einstieg für die Arbeitgeber sein [18]. Die Qualität der SDB hat sich in den letzten Jahren erfahrungsgemäß durch die belastbaren Daten aus den REACH-Registrierungen verbessert.

Allerdings sind die SDB von Stoffen und Gemischen jetzt auch erheblich umfangreicher. Einige Informationen sind nicht ausreichend auf die Risikobewertung im Betrieb angepasst oder überflüssig, andere könnten ergänzt werden, wie z. B. die eindeutige Angabe eines Maßnahmenniveaus, wie es im Einfachen Maßnahmenkonzept Gefahrstoffe (EMKG) erfolgt (s. Abschnitt zum EMKG unten). Die BAuA erforscht die Bedürfnisse der Anwender in dem Projekt REACh2SDS [19]. Dabei geht es um die Datenverfügbarkeit und -qualität vom Registrierungsdossier über das SDB bis hin zu ihrem Nutzen bei der Risikobewertung am Arbeitsplatz. Auch der Verband der chemischen Industrie (VCI) hat eine Analyse durchgeführt und die Erkenntnisse zur Anwendbarkeit der SDB in einem Bericht veröffentlicht [20, 21]. Innerhalb der aktuell geplanten REACH-Revision wären die Berücksichtigung dieser Ergebnisse und die stärkere Einbindung von Personen, die Risikobewertungen im Betrieb durchführen, wünschenswert [22].

### Digitalisierung von Sicherheitsdatenblättern

Die sicherheitsrelevanten Informationen der Hersteller oder Importeure chemischer Stoffe werden häufig über einen langen Weg entlang der Lieferkette von Lieferant zu Lieferant und letztendlich zum Endanwender weitergegeben. Meistens erfolgt die Weitergabe der SDB als Papierausdruck oder im PDF-Format. Informationen können nur mühsam herausgesucht und händisch übertragen werden. Es besteht zusätzlich die Gefahr, dass Informationen entlang der Kommunikationskette verfälscht werden oder verloren gehen.

Ein harmonisiertes digitales Format könnte hier Abhilfe schaffen. Viele Akteure in der Lieferkette wünschen sich ein derartiges Format, das einen digitalen Datentransfer und eine innerbetriebliche Datenverarbeitung ermöglicht. Im 2018 veröffentlichten 3. Bericht der Europäischen Kommission zur Umsetzung der REACH-Verordnung wurde die Notwendigkeit für diese Entwicklungen erkannt [22]. Inzwischen ist es ein zentraler Punkt im Arbeitsplan des von der Europäischen Chemikalienagentur (ECHA) eingesetzten Netzwerkes für den Austausch von Expositionsszenarien [23].

Für die chemische Industrie und die Bauwirtschaft wurde im Projekt „SDBtransfer“ erstmals ein durchgängiger Prozess für den elektronischen Austausch von sicherheitsrelevanten Daten entwickelt. Die Ergebnisse zeigen eindeutig Vorteile für alle Akteure in der Lieferkette auf [24]. Mit dem standardisierten elektronischen Austausch von SDB beschäftigen sich ebenfalls der Bundesverband der Deutschen Industrie (BDI) und der Verband der europäischen chemischen Industrie (Cefic; [25]).

### Grenzwerte als Hilfsmittel zur Beurteilung der Exposition

Neben der Gefahrstoffeigenschaft ist auch die zweite Säule der Risikobewertung, die Exposition der Beschäftigten gegenüber Gefahrstoffen, zu bestimmen. Um die Exposition gegenüber einem Gefahrstoff zu bewerten, werden Grenzwerte als Beurteilungsmaßstäbe abgeleitet. Im Allgemeinen gilt, je niedriger der Grenzwert, desto höher die potenzielle Gefahr, die vom Gefahrstoff ausgeht.

Neben Grenzwerten für die Luftbelastung am Arbeitsplatz (Arbeitsplatzgrenzwerte) gibt es Grenzwerte für die innere Belastung, die Auskunft darüber geben, wie hoch die Konzentration von Gefahrstoffen oder ihren Metaboliten im Körper ist (biologische Grenzwerte). Über deren Messung besteht bei der arbeitsmedizinischen Vorsorge die Möglichkeit, eine Gefahrstoffaufnahme zu erfassen. Beiden Arten von Grenzwerten ist gemeinsam, dass sie gesundheitsbasiert sind. Auf Grundlage von Tierversuchen und epidemiologischen Studien wurde ermittelt, dass gesunde erwachsene Beschäftigte bei der Einhaltung der Grenzwerte keinem erhöhten Risiko ausgesetzt sind und nicht erkranken.

Liegen Grenzwerte für einen Gefahrstoff vor, sind Arbeitgeber verpflichtet, sie einzuhalten. In Deutschland sind zurzeit mehr als 450 Arbeitsplatzgrenzwerte in der Technischen Regel für Gefahrstoffe (TRGS) 900 und ca. 60 biologische Grenzwerte in der TRGS 903 aufgelistet [26, 27]. Allen Grenzwerten ist gemeinsam, dass auf Grundlage wissenschaftlicher Daten eine Schwelle abgeleitet werden kann, unterhalb derer für gesunde Beschäftigte keine Gesundheitsgefahr besteht (Schwellenwertstoffe).

**Figure 4:**
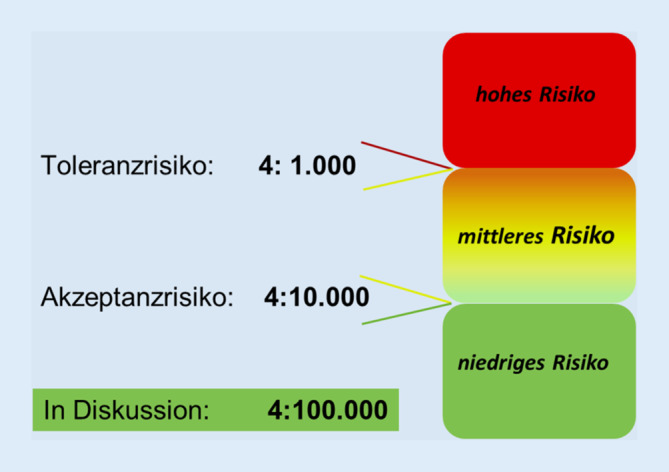

Daneben gibt es aber eine Reihe von Gefahrstoffen, bei denen *keine *Schwelle abgeleitet werden kann. Dies betrifft vor allem krebserzeugende Gefahrstoffe. Hier verbleiben auch bei niedriger Exposition Restrisiken einer Krebsentstehung, auch wenn diese für Beschäftigte sehr niedrig sind. Um hier die Praxis zu unterstützen, hat der Ausschuss für Gefahrstoffe (AGS) ein Risikokonzept für krebserzeugende Gefahrstoffe entwickelt ([28]; Abb. 4). Grundlage ist ein intensiv geführter Diskurs aller Stakeholder, an dessen Ende ein Konsens über akzeptierte und tolerierte Risikogrenzen erzielt wurde. Das Akzeptanzrisiko entspricht einem zusätzlichen Krebsrisiko von 4:10.000, d. h., dass statistisch von 10.000 während des gesamten Arbeitslebens exponierten Personen 4 an Krebs erkranken. Das Toleranzrisiko entspricht 4:1000. Weitere Informationen dazu enthält die TRGS 910 [29].

Mit diesem Konzept lassen sich *risikobasierte* Grenzwerte ableiten, eine Akzeptanz- und eine Toleranzkonzentration. Risiken werden über stoffspezifische Risikobereiche transparent ausgewiesen und mit einem gestuften Maßnahmenkonzept verknüpft. Liegt die Expositionshöhe im oder in der Nähe des Rotbereichs, sind dringend geeignete Maßnahmen zum Schutz der Beschäftigten zu ergreifen. Im Grünbereich sind hingegen keine zusätzlichen Arbeitsschutzmaßnahmen zwingend erforderlich.

Orientierung für die Festlegung von Akzeptanz- und Toleranzgrenzen boten Krebsrisiken aus anderen Lebensbereichen (z. B. spontane Krebserkrankungen, UV-Strahlung). Mehr als 12 Jahre Erfahrung mit dem Risikokonzept für Kanzerogene haben gezeigt, dass die Kommunikation dieses Konzeptes in Deutschland für mehr Transparenz von Krebsrisiken am Arbeitsplatz gesorgt hat. Daher ist es Ziel der BAuA, das Konzept in gleicher oder ähnlicher Form auch im europäischen Gefahrstoffrecht zu verankern [30].

### Technische Regeln für Gefahrstoffe zur Beschreibung sicherer Arbeitsbedingungen

Technische Regeln für Gefahrstoffe (TRGS) haben verschiedene Funktionen. Dazu gehören die Unterstützung der Arbeitgeber bei Tätigkeiten mit hohen Risiken durch Gefahrstoffe, der Schutz vor Brand und Explosion, die Messung von Gefahrstoffen und die Substitutionsprüfung.

Das fachliche Niveau von TRGS ist im Allgemeinen hoch. Rechtlich haben sie den Status eines vorweggenommenen Sachverständigengutachtens, weil sie im Konsens der Arbeitsschutzexperten im AGS entwickelt wurden. Sie entfalten die „Vermutungswirkung“, d. h., wenn ein Betrieb die Maßnahmen einer TRGS umsetzt, gelten gegenüber den Aufsichtsdiensten die Anforderungen der Gefahrstoffverordnung als erfüllt. Obwohl es seit rund 50 Jahren TRGS gibt, sind den Autoren nur 2 Fälle bekannt, bei denen gegen Inhalte von TRGS (zurzeit ca. 70) Klage vor Verwaltungsgerichten erhoben wurden. Beide Fälle liegen mehr als 20 Jahre zurück [31].

Ein Betrieb kann aber auch eigene Lösungen entwickeln. Dann muss er gegenüber den Aufsichtsdiensten nachweisen, dass seine Lösungen mindestens genauso wirksam sind, wie die in einer TRGS beschriebenen. Wegen des rechtlichen Status haben TRGS im nationalen Gefahrstoffrecht und darüber hinaus eine hohe Bedeutung. Auf der anderen Seite führt der hohe Anspruch an TRGS oft zu umfangreichen Regeln, die teilweise schwer verständlich sind. Besonders kleinste, kleine und mittlere Unternehmen (KKMU) haben Schwierigkeiten, einzelne TRGS umzusetzen. Sie benötigen ein weiteres Format, das an ihre Bedürfnisse angepasst ist.

## Adressatengerechte Risikokommunikation an die betrieblichen Akteure und die breite Öffentlichkeit

### Unterschiedliche Voraussetzungen der Adressaten

Die Akzeptanz für ein risikoorientiertes Handeln kann gefördert werden, wenn die Risikokommunikation adressatengerecht erfolgt. In einem Großunternehmen reichen die zur Verfügung gestellten Instrumente des Lieferanten, des Gesetzgebers und der Unfallversicherungsträger in der Regel aus. Fachleute, wie z. B. Sicherheitsfachkräfte und Betriebsärzte und -ärztinnen, verfügen über die notwendige Kompetenz und den Handlungsspielraum, anhand dieser Instrumente Risiken im Betrieb zu erkennen und die Unternehmensführung bezüglich geeigneter Schutzmaßnahmen zu beraten.

Eine „Übersetzung“ einer TRGS in weitere Handlungsempfehlungen, wie es z. B. die Unfallversicherungsträger, die Bundesländer, die BAuA, Verbände oder andere Interessensvertreter praktizieren, ist für die Risikokommunikation in KKMU essenziell. In Deutschland sind 96 % aller Unternehmen kleinste und kleine Unternehmen mit weniger als 50 Beschäftigten [32]. Instrumente der Risikokommunikation für KKMU sind erfolgreich, wenn sie an die Erfahrungswelt und Ausbildung der Verantwortlichen anknüpfen. Die Ausbildung kann je nach Größe des Betriebs und der Branche sehr unterschiedlich sein [17]. Die Inhalte dieser Instrumente sollten einen Einstieg unterhalb des technischen Regelwerks bieten, leicht verständlich und an die Sprache der Branche angepasst sein. Gerade in kleinsten und kleinen Unternehmen, bei denen der Inhaber einer Firma aktiv in die Arbeitsprozesse eingebunden ist, sollten Schritte so definiert sein, dass ein leichter Einstieg in die Risikobewertung möglich ist.

### Einfaches Maßnahmenkonzept Gefahrstoffe – EMKG

Seit 2005 stellt die BAuA mit dem „Einfachen Maßnahmenkonzept Gefahrstoffe“ (EMKG) Instrumente zur Risikokommunikation für KKMU bereit [33–37]. Auch Anwender mit wenigen Kenntnissen des Gefahrstoffrechts können mit dem EMKG Risiken systematisch und mit wenig Aufwand bewerten.

Für die Einschätzung des Risikos werden leicht zugängliche Informationen aus dem Sicherheitsdatenblatt und Angaben zur Tätigkeit benötigt. Das Ergebnis ist jeweils eine Maßnahmenstufe für die Module *Haut, Einatmen* sowie *Brand und Explosion*. Je höher das Risiko, desto höher die Maßnahmenstufen. Diese sind mit konkreten Schutzleitfäden zur Gestaltung der Arbeitsverfahren verknüpft. Ein Teil der Schutzleitfäden ist mit Videosequenzen hinterlegt, die anschaulich die Erhöhung der Exposition, z. B. durch falsche Positionierung einer Absaugung, demonstrieren [38].

**Figure 5:**
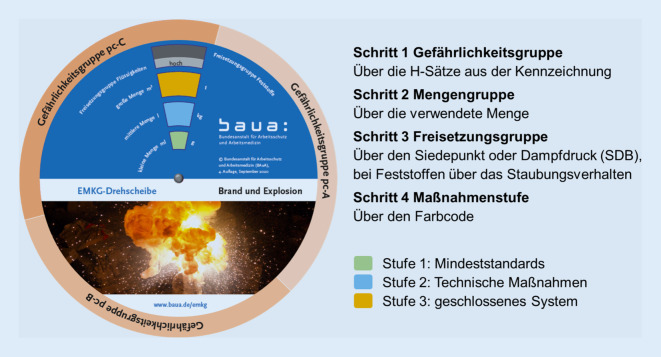

Konzeptionell ist das EMKG in 2 Leitfäden beschrieben. Als Argumentationshilfen zur Kommunikation der Risiken wurden EMKG-Drehscheiben (Abb. 5), eine Smartphone-App, eine PC-Software und ein EMKG-Poster erstellt, die direkt am Arbeitsplatz einsetzbar sind. Im Jahresdurchschnitt wurden die Leitfäden, Poster und Drehscheiben (Printversion oder Download) jeweils in einer Größenordnung von 5000 angefordert.

In einem Projekt der BAuA zur Entwicklung des EMKG 3.0 wurde ein Didaktikkonzept gestaltet. Im Projektverlauf wurde festgestellt, dass die anvisierte Zielgruppe KKMU nur bedingt erreicht wurde. Anhand einer Analyse der Bedürfnisse von KKMU wurden didaktische Leitlinien beschrieben, um diese Situation zu verbessern. In den letzten Jahren wurden die EMKG-Produkte entsprechend angepasst und neue Verbreitungswege etabliert. Ein wichtiger Aspekt hierbei war eine stärkere Verknüpfung zwischen Ausbildungsinhalten und dem EMKG [39]. In der Ausbildung zur Sicherheitsfachkraft ist das bereits gelungen [40], in der Berufs- und Meisterausbildung besteht noch Handlungsbedarf.

### Spezielles Training für Tätigkeiten mit Diisocyanaten

Ein neuer Weg, chemische Risiken adressatengerecht zu kommunizieren, wird zurzeit auf europäischer Ebene beschritten. Grundsätzlich sind Arbeitgeber nach der Gefahrstoffverordnung verpflichtet, ihre Beschäftigten zu unterweisen. Neu sind Verpflichtungen im Hinblick auf die Inhalte und den Umfang der Unterweisung. Wegweisend ist die Beschränkung für die Vermarktung von Diisocyanaten unter der REACH-Verordnung [6]. Diisocyanate werden in vielfältigen Anwendungen und großen Mengen (> 2,5 Mio. t/a) für die Produktion von Polyurethanschaumstoffen eingesetzt (z. B. in Möbeln, auf dem Bau, in der Automobilproduktion). Etwa 5 Mio. Beschäftigte sind EU-weit exponiert und durch Erkrankungen an schwerem Asthma und Hautallergien gefährdet (ca. 6500 Fälle im Jahr). Trotz europäischer und nationaler Arbeitsschutzregelungen ist seit 2005 nur noch ein minimaler Rückgang bei den Erkrankungen zu beobachten.

Daraus haben die EU-Kommission und die Mitgliedstaaten geschlossen, dass die derzeitigen Maßnahmen nicht ausreichen, die Beschäftigten adäquat zu schützen. Mit der Beschränkung wurde entschieden, dass Diisocyanate vom Hersteller nur dann an die Verwender (weiterverarbeitenden Betriebe) verkauft werden dürfen, wenn die Verwender nachweisen, dass ihre Beschäftigten für die spezifische Anwendung im Betrieb entsprechend geschult worden sind [41]. Die Verknüpfung eines Markteingriffs (der Beschränkung) mit verpflichtenden Schulungen für die Beschäftigten (eine Maßnahme des Arbeitsschutzes) ist ein innovativer Ansatz der Chemikalienregulation. Um Diisocyanate zu erwerben, muss das spezifische Training gegenüber dem Hersteller nachgewiesen werden. Beschäftigte sind verpflichtet, an den Trainings teilzunehmen. Die europäischen Herstellerverbände entwickeln die Trainingsinhalte und stellen diese in 24 Sprachen der EU zur Verfügung [42].

In einer epidemiologischen Längsschnittstudie soll in den nächsten Jahren die Wirksamkeit dieses neuen Regelungsansatzes zum Schutz der Beschäftigten evaluiert werden [43].

### DASA Arbeitswelt Ausstellung

Ein anderer Weg zur Kommunikation von Risiken kann anhand von Objekten erfolgen. Diesen Weg geht die „DASA Arbeitswelt Ausstellung“ in Dortmund. Sie wurde 1993 unter dem Namen „Deutsche Arbeitsschutzausstellung“ eröffnet. Sie versteht sich als kreativer Lernort für Sicherheit und Gesundheit bei der Arbeit für die breite Öffentlichkeit, insbesondere für Schulklassen in der Berufsorientierungsphase. In dem Bezugsrahmen Mensch – Arbeit – Technik vermittelt sie wissenschaftsbasiert Arbeitswelten der Vergangenheit, Gegenwart und Zukunft in Form einer Dauerausstellung und in Wechselausstellungen. Das Erreichen der Ziele wird durch regelmäßige Besucherforschung qualitätsgesichert. Beim Thema Gefahrstoffe wird beispielhaft das im Arbeitsschutz geltende STOP-Prinzip (Substitution vor Technik vor Organisation vor persönlicher Schutzausrüstung) erklärt [44]. Danach hat beispielsweise die Gestaltung des Arbeitsverfahrens, wie etwa die Absaugung von Gefahrstoffen oder organisatorische Maßnahmen zur Kontaktvermeidung, Vorrang vor der persönlichen Schutzausrüstung. Die beste Lösung besteht jedoch darin, wenn möglich, die Gefahrstoffe durch weniger gefährliche zu ersetzen oder das Risiko durch geeignete Verfahren wie z. B. geschlossene Systeme zu minimieren.

Ein gutes Beispiel für Ersatzstoffe wird an einer Offsetdruckmaschine erzählt: Bereits in den späten 1980er-Jahren haben skandinavische Drucker mit Lebensmittelölen als Alternative zu den gefährlichen lösemittelhaltigen Reinigern experimentiert. Heute gibt es eine ganze Reihe von erprobten Ersatzstoffen im Druckgewerbe. Hightechprodukte der chemischen Industrie, die mit den Salatölen der ersten Zeit nur noch die Idee verbindet, werden aus nachwachsenden Rohstoffen gewonnen. Dort, wo sie eingesetzt werden, hat sich die Exposition gegenüber Lösemitteln in Druckbetrieben drastisch verringert – ein gutes Beispiel für die Ziele der aktuellen EU-Chemikalienstrategie für Nachhaltigkeit [45].

## Diskussion

Die Informationen für eine adäquate Risikobewertung und damit für eine passgenaue Kommunikation von Risiken beim Umgang mit Gefahrstoffen im Betrieb sind durch die Verpflichtungen der europäischen Chemikalienregelungen (CLP, REACH) vorhanden. Die Qualität der Sicherheitsdatenblätter, dem wichtigsten Instrument der Risikokommunikation, hat sich durch die REACH-Verordnung in Bezug auf belastbare Daten verbessert. Gleichzeitig hat sich jedoch der Umfang der Sicherheitsdatenblätter erheblich vergrößert. Daher wünschen sich die Autoren eine Reduktion der Informationsfülle auf das Wesentliche und damit eine bessere Anpassung an die Bedürfnisse der Risikobewerter im Betrieb.

Auf Ebene der Gefahrstoffverordnung unterstützen technische Regeln für Gefahrstoffe die Unternehmen und geben durch ihre Vermutungswirkung Rechtssicherheit. Mit ihrem hohen fachlichen Anspruch haben sie sich für Fachleute im Arbeitsschutz bewährt. Technische Regeln sind aber besonders für KKMU häufig zu umfangreich und schwer verständlich. Das Einfache Maßnahmenkonzept Gefahrstoffe (EMKG) und die passenden Schutzleitfäden haben sich unterhalb des Niveaus technischer Regeln etabliert, um die Kommunikation von Risiken in KKMU zu verbessern und Arbeitgeber zu risikoorientiertem Handeln zu motivieren.

## Ausblick

Erste Untersuchungen zeigen, dass die Informationen im Sicherheitsdatenblatt nicht immer an die Bedürfnisse der Personen angepasst sind, die im Betrieb Risiken analysieren und bewerten. Deshalb plant die BAuA in der aktuell stattfindenden REACH-Revision diesen Aspekt verstärkt in die Diskussionen einzubringen. Zusätzlich soll der Datentransfer in Richtung einheitliches digitales Sicherheitsdatenblattformat auf den Weg gebracht werden. Wichtige Informationen für die Risikoanalyse und zu Schutzmaßnahmen können dann sofort in die Risikobewertung und in die Betriebsanweisung übernommen werden. Weiteren Handlungsbedarf sieht die BAuA in der Unterstützung von KKMU. Auf Grundlage von positiven Erfahrungen mit der Ausbildung von Sicherheitsfachkräften ist die Integration von Inhalten des EMKG in die Berufs- und Meisterausbildung ein möglicher Weg. Weiterhin ist nach einer inzwischen mehrjährigen Erprobung in der Praxis geplant, das Didaktikkonzept des EMKG zu evaluieren, um ggf. Anpassungsbedarf für KKMU zu ermitteln.
